# Sensorimotor Learning in Response to Errors in Task Performance

**DOI:** 10.1523/ENEURO.0371-21.2022

**Published:** 2022-03-16

**Authors:** Dhwani P. Sadaphal, Adarsh Kumar, Pratik K. Mutha

**Affiliations:** 1 Center for Cognitive and Brain Sciences, Indian Institute of Technology Gandhinagar, Palaj, Gandhinagar 382355, India; 2 Department of Mechanical Engineering, Indian Institute of Technology Gandhinagar, India; 3 Department of Biological Engineering, Indian Institute of Technology Gandhinagar, Palaj, Gandhinagar 382355, India

**Keywords:** goal-directed control, motor learning, performance errors, stimulus-response learning, strategies

## Abstract

The human sensorimotor system is sensitive to both limb-related prediction errors and task-related performance errors. Prediction error signals are believed to drive implicit refinements to motor plans. However, an understanding of the mechanisms that performance errors stimulate has remained unclear largely because their effects have not been probed in isolation from prediction errors. Diverging from past work, we induced performance errors independent of prediction errors by shifting the location of a reach target but keeping the intended and actual kinematic consequences of the motion matched. Our first two experiments revealed that rather than implicit learning, motor adjustments in response to performance errors reflect the use of deliberative, volitional strategies. Our third experiment revealed a potential dissociation of performance-error-driven strategies based on error size. Specifically, behavioral changes following large errors were consistent with goal-directed or model-based control, known to be supported by connections between prefrontal cortex and associative striatum. In contrast, motor changes following smaller performance errors carried signatures of model-free stimulus-response learning, of the kind underpinned by pathways between motor cortical areas and sensorimotor striatum. Across all experiments, we also found remarkably faster re-learning, advocating that such “savings” is associated with retrieval of previously learned strategic error compensation and may not require a history of exposure to limb-related errors.

## Significance Statement

Humans adjust their actions if they do not produce desired limb-related sensory consequences or task-related outcomes. We probed whether task-related performance errors induce implicit changes to motor plans at all, or simply trigger the deliberate selection of different actions. We induced performance errors in isolation, and found that they were compensated entirely via intentional, strategic mechanisms consistent with improved action selection. Strategies also appeared to be sensitive to error size, and transitioned from stimulus-response associative behavior to goal-directed control as error magnitude increased. Across all experiments, we also found faster re-learning or “savings,” substantiating the view that savings is associated with strategy-use, and does not depend on experience of limb-related prediction errors that bring about implicit adjustments to action plans.

## Introduction

Studies of motor adaptation, the capacity to recalibrate our actions to changing body and environmental conditions, have been instrumental in characterizing many fundamental principles of sensorimotor learning. Adaptation paradigms have typically employed different visual (Scheidt et al., 2005; Morehead et al., 2017) or dynamic (Shadmehr and Mussa-Ivaldi, 1994; Sainburg et al., 1999; Lefumat et al., 2015) perturbations that produce discrepancies in the actual versus expected limb-related sensory feedback. It is generally believed that such sensory prediction errors (SPEs) are compensated by implicitly recalibrating motor plans (Mazzoni and Krakauer, 2006; Morehead et al., 2017; Oza et al., 2020). SPE-driven changes in motor output are dependent on cerebellar (Flament et al., 1996; Martin et al., 1996; Morehead et al., 2017) and posterior parietal networks (Clower et al., 1996; Della-Maggiore et al., 2004; Kumar et al., 2020); disruption in these regions, either naturally because of Stroke or degeneration, or artificially using brain stimulation techniques, produces clear deficits in SPE-based learning.

Perturbations applied to moving effectors produce not just SPEs, but can also result in task performance errors (TPEs). In goal-directed motion, TPEs could arise from a failure to achieve the movement goal (missing a spatial target, for instance), or when a target moves to a different location while the action is being performed. Learning to compensate TPEs plausibly requires intact cortico-striatal circuits (Anguera et al., 2010; Taylor and Ivry, 2012), although a measure of the TPE itself could come from the simple spike discharge of cerebellar Purkinje neurons (Popa et al., 2017). However, a clear understanding of the computational and psychological mechanisms that drive changes in motor behavior on exposure to recurring TPEs, has remained elusive. While early work hinted that TPEs may not induce an implicit adaptive response, it did not elaborate on the algorithms employed (Diedrichsen et al., 2005). Later studies suggested that TPEs could provoke use of deliberative movement re-aiming strategies (Taylor et al., 2014; McDougle and Taylor, 2019), but an alternative proposition has been put forth in more recent work. This latter set of studies, which have probed the influence of binary TPEs on learning, suggests that like SPEs, TPEs can drive implicit learning, and net adaptation reflects the sum of two implicit processes, one driven by SPE and the other by TPE (Leow et al., 2018; Van der Kooij et al., 2018; Kim et al., 2019). These two views thus differ in terms of how TPEs contribute: one suggests that they drive the formulation of an explicit strategy, while the other invokes implicit recalibration.

This debate arises primarily because TPEs have rarely been elicited independent of SPEs. When these errors co-occur, it is likely that they interact, which, neuroanatomically, could be facilitated via connections between the basal ganglia and the cerebellum (Bostan and Strick, 2018). Furthermore, this interaction may be competitive, with SPEs dominating the adaptative response (Wang et al., 2019). This is supported by findings in healthy individuals who adapt to SPEs even if it amplifies TPEs (Mazzoni and Krakauer, 2006), or who show SPE-driven learning even though they cannot correct for TPEs because of task constraints (Tseng et al., 2007). Likewise, Stroke patients with lesions circumscribed to right inferior frontal cortex show complete adaptation to SPEs despite failing to correct for TPEs (Mutha et al., 2011). Given this overwhelming influence of SPEs when imposed concurrently with TPEs, it is perhaps not surprising that mechanisms through which TPEs alone are compensated have remained unclear.

Resolving the mechanisms underlying TPE-mediated changes in motor behavior also has implications for understanding the formation of long-term motor memories. Such latent memories enable faster learning on re-exposure to the perturbation, a phenomenon termed “savings.” While there is evidence that savings is promoted via strategic re-aiming (Haith et al., 2015; Huberdeau et al., 2015; Morehead et al., 2015), some studies have linked it to other processes including implicit mechanisms (Coltman et al., 2019; Yin and Wei, 2020), action repetition (Huang et al., 2011), and a memory of the experienced errors that in turn modulates error sensitivity (Herzfeld et al., 2014). Based on these diverse results, one cannot be certain whether it is improved action selection (mediated by TPEs) or improved action execution (mediated by SPEs) or a combination of the two that contributes to long-term motor memory formation that facilitates savings.

Here, we examined how humans learn to compensate consistent TPEs imposed in isolation from SPEs, and whether they express as savings the acquired memory when re-exposed to the learning environment. We also probed whether and how the magnitude of the TPE influences the ensuing changes in motor output.

## Materials and Methods

### Subjects

We recruited 76 healthy, right-handed individuals between the age 18 and 30 years across three different experiments. Handedness was assessed using the Edinburgh Handedness Inventory. All subjects were naive to the expected outcomes of the experiment, provided written informed consent before participating, and were paid for their time. The study was approved by the Institute Ethics Committee of the Indian Institute of Technology Gandhinagar. One subject was excluded (see below), resulting in a total of 75 subjects (mean age = 22.64 ± 0.34 years, 27 females) whose data were analyzed.

### Experimental setup

**Figure 1.**
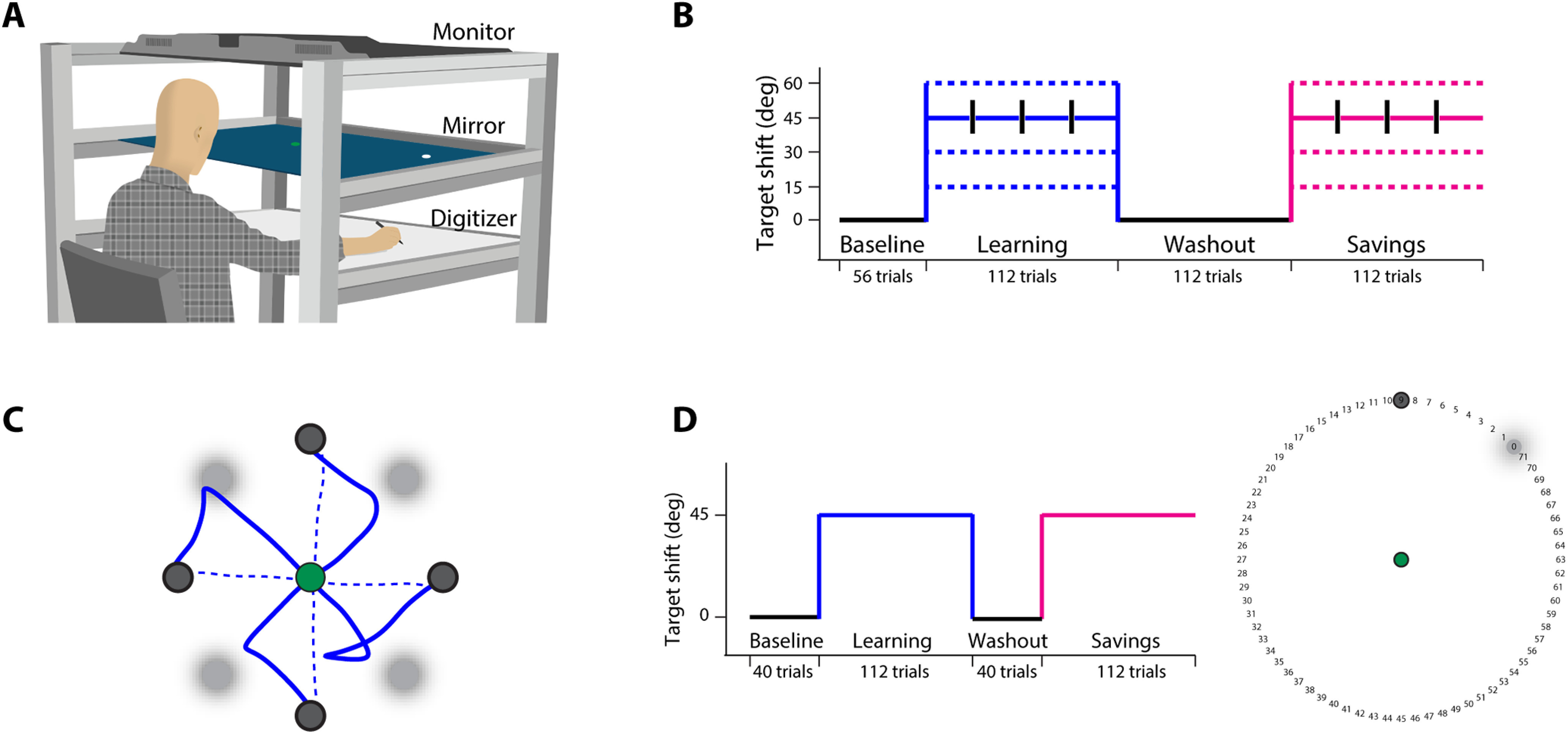
Experimental setup and tasks. ***A***, Subjects performed reaching movements on a digitizing tablet using a handheld stylus while looking into a mirror placed between the tablet and a horizontally mounted display. Start positions, targets, and a feedback cursor displayed on the screen were reflected in the mirror. ***B***, Task protocol for experiments 1 and 3. The baseline block was followed by learning trials on which the target-shift created a TPE. This was followed by washout and a final “savings” block on which subjects re-experienced the target-shifts. In experiment 1, the target-shift was 45° (solid line), while in experiment 3, it was 15°, 30°, or 60° (dotted lines) for different groups. In both experiments, three “no-shift” sub-blocks of four trials each were embedded during learning and savings trials; their location is shown using black bars. Verbal instructions were given every time the target conditions were about to change. ***C***, Target locations and sample hand trajectories on early (solid) and late (dotted) learning trials. The original target has been blurred, while the new, shifted target is shown in solid colors. ***D***, In experiment 2, subjects again performed four blocks of trials, but without the no-shift sub-blocks. Additionally, the original target was presented with a ring of numbers as shown on the right. Before each trial, subjects reported the approximate number they would reach to. The original target location always corresponded to number “0,” while the shifted target corresponded to “9.” The ring appeared with the original target and disappeared with the presentation of the new target.

Subjects sat on a height-adjustable chair facing a large, horizontally placed digitizing tablet and used a hand-held stylus to make planar, targeted reaching movements on it (Fig. 1*A*). All movements were made with the right hand. Subjects received visual feedback of their hand (stylus) position on a mirror that reflected a high-definition display placed directly above it. The mirror was aligned parallel to the screen and the digitizing tablet, and prevented direct view of the moving limb. Hand position was displayed as a circular cursor (0.5 cm diameter) along with a circular start position (1 cm diameter) and targets (1.5 cm diameter) for the reach.

To begin a trial, subjects first brought the cursor into the start circle. After a delay of 500 ms, a target appeared at one of four locations (45°, 135°, 225°, or 315°) along with an audio beep that indicated to subjects that they should start moving. Across all experiments, the distance between the start position and the target was fixed at 10 cm, and subjects were encouraged to move as quickly as possible, but no specific constraints were imposed on either reaction time (RT) or movement time. Further, cursor feedback was provided during the entire reach and was always veridical with the actual position of the hand.

### Experimental blocks

In all three experiments of this study, subjects performed four blocks of trials: baseline, learning, washout, and savings. Baseline trials comprised of reaches to fixed, stationary targets. This was followed by the learning block in which the target location was shifted, or “jumped,” counterclockwise on each trial. The shift was achieved by extinguishing the originally displayed target (“original” target) and immediately displaying a new one (“new” target). The magnitude of the target-shift was 45° in experiments 1 and 2, while it was 15°, 30°, or 60° for the different groups of experiment 3 (Fig. 1*B*,*D*; also see below). The shift was initiated as soon as subjects breached the start circle boundary (moved 3 mm from the center of the start circle), and enabled us to impose a TPE. The learning block, in which subjects learned to predictively account for the TPE (Fig. 1*C*), was followed by washout trials that were similar to baseline in that there was no target-shift, and the original target remained on the screen for the entire trial. After washout, we probed for “savings” by exposing subjects to target-shifts as in the earlier learning block. Specific task instructions were given before the onset of each block (see below).

To gain some familiarity with the setup and the task display, subjects first performed 10 no-shift trials and then two target-shift trials; these 12 practice trials were not analyzed. Before they attempted the practice no-shift trials, subjects were explained what they would see on the screen and told that they should reach from the start circle to the target. Before the practice target-shift trials, they were told that they might experience trials in the task where the target would jump to a different location. They then performed two such trials as practice. Throughout the experiment including practice trials, at the end of each trial, subjects were given points (10, 5, 3, or none) depending on the accuracy of their movement. Accuracy was calculated relative to the original target on baseline and washout trials, and the new target on the learning and savings trials. Points were not analyzed.

### Experiment 1

In our first experiment (*n* = 30), subjects performed 56 baseline, 112 learning, 112 washout, and 112 savings trials. As described earlier, baseline and washout trials comprised of reaches to stationary targets, while the target was shifted 45° counterclockwise during the learning and savings blocks. Additionally, interspersed within the learning and savings blocks were three sub-blocks of four trials each on which the target was not shifted; these trials were thus similar to baseline (Fig. 1*B*) and did not induce a TPE. Each of the no-shift sub-blocks occurred after every 28 target-shift trials. In all, subjects performed 416 trials in this first experiment.

Subjects were given verbal instructions before each of the main experimental blocks and also before each no-shift sub-block embedded within the learning and savings blocks. Before the baseline block, subjects were told to reach to the target that would be displayed on the screen, and were also informed that its position would not change. Following baseline and before the onset of the learning block, subjects were told that the target would now start “jumping,” and that they should reach to the new target. Further, before each no-shift sub-block, they were told that the target would now stop jumping and they should move to the original target. Similarly, at the end of each no-shift sub-block, subjects were informed that the target would start jumping again and they should go to the new target. Instructions before the washout block were similar to those given before the no-shift sub-blocks. Instructions provided before the savings block were the same as those given before the learning block. In sum, verbal instructions were given every time the target-shift conditions were about to change.

### Experiment 2

The design of our second experiment (*n* = 10) was motivated by the work of Taylor et al. (2014), who used verbal reports of subjects’ intended aiming direction to estimate their use of cognitive strategies. The setup and general task environment remained similar to that of experiment 1. Subjects performed 40 baseline, 112 learning, 40 washout, and 112 savings trials. The reach target remained stationary on the baseline and washout trials, while 45° counterclockwise target shifts were introduced on each trial of the learning and savings blocks (Fig. 1*D*). Target presentation and timing of the jump remained similar to experiment 1. The no-shift sub-blocks were not employed in this experiment.

In addition to the start circle and the target, a ring of 72 numerical landmarks (numbered from 0 to 71, increasing counterclockwise) placed at 5° intervals along the periphery of a virtual circle of 10 cm diameter (corresponding to the target distance) was also presented on each trial of all four blocks (Fig. 1*D*). Since the target could appear at any one of four different locations, the ring was rotated such that landmark “0” always coincided with the location of the original target for that trial while landmark “9” always corresponded to the location of the new target displayed on learning and savings trials (45° counterclockwise). The ring was presented simultaneous with the original target and it disappeared once the subjects crossed the edge of the start circle. Importantly, on every trial, before they initiated their movement, subjects were required to verbally report their aiming direction by stating the approximate numerical landmark they intended to move to. This number was recorded by the experimenter.

As in experiment 1, subjects were also informed about target behavior before each block. Briefly, before baseline trials, subjects were told that they should move to the target that would be displayed on the screen, and that its location would not change during the trial. Before the learning block, subjects were informed that the target would now start “jumping” during the trial and they should reach to the new target. Before washout, they were again informed that the target would stop jumping and they should move to the original target. Finally, before the savings block, they were told that the target would start jumping again and they should go to the new target.

### Experiment 3

In experiment 3 (*n* = 36, one subject was excluded from the analysis, so final *n* = 35), we aimed to understand the influence of TPE magnitude on changes in motor behavior. Subjects were assigned to three different groups, that differed in terms of the magnitude of the target-shift experienced [15° (*n* = 11), 30° (*n* = 12), or 60° (*n* = 12)]. All shifts were counterclockwise as before, and all other aspects of this experiment were identical to experiment 1 (Fig. 1*B*). Thus, subjects performed four blocks: baseline (56 trials), learning (112 trials), washout (112 trials), and savings (112 trials). Targets remained stationary during the baseline and washout blocks, while they were shifted on learning and savings trials. Three no-shift sub-blocks (four trials each) were also embedded within the learning and savings blocks. Instructions to subjects and their schedule remained the same as in experiment 1.

### Data analysis

#### Variables

Data were analyzed using custom MATLAB scripts. Hand X and Y position data were filtered using a low-pass Butterworth filter with 10 Hz cutoff. Position data were differentiated to obtain the speed profile. Movement onset was defined as the point at which hand speed first crossed 5% of maximum movement speed. Reaction Time (RT), a variable of interest in experiments 1 and 3, was calculated as the difference between the time of movement onset and the time of target presentation. Our other key measure was the deviation in hand movement direction relative to the direction of the original target. This was calculated as the angle between two lines: the line joining the center of the start circle and the original target, and the line joining the center of the start circle and the hand position at peak speed. On a few trials, more than one peak could occur. For example, on the early learning trials (Fig. 1*C*), subjects could make an initial outward movement to the original target and then correct it online to go to the new target, resulting in two peaks in the speed profile. In such cases, hand position at the first large (>15 cm/s) peak, corresponding to the outward movement to the original target, was chosen for the calculation of hand deviation since this would serve as a more appropriate indicator of the subjects’ initial movement plan. Counterclockwise and clockwise deviations relative to the original target were treated as positive and negative, respectively.

#### Outlier removal

First, trials on which subjects did not move, or moved but lifted the stylus off the digitizing tablet leading to data loss, were marked as bad trials. Second, outliers were identified based on the hand deviation data. For the baseline and washout blocks, we first calculated the mean hand deviation across all trials of that block, and then labeled as an outlier any trial on which the hand deviation was more than ±3 SDs from the corresponding mean. For the learning and savings blocks, outliers were marked as those trials on which the hand deviation was more than ±3 times the magnitude of the target-shift. Following this procedure, one subject from the 15° jump group of experiment 3 ended up with 136 bad/outlier trials (out of 416 trials performed); this subject was excluded entirely. Across all the remaining 75 subjects, 1.34% of the trials were labeled as bad trials or outliers and removed from the analysis.

#### Further analyses and statistics

Following outlier removal, potential baseline biases in reach direction were eliminated by subtracting the mean baseline hand deviation from the hand deviation on each trial; these baseline-subtracted values were used for further analyses. Average hand deviation and RT on the last twelve baseline trials were taken as an indicator of late baseline behavior. We also computed the mean hand deviation and RT on the first and last unique reaches to each target (four trials) of the learning, washout and savings blocks. This provided a measure of early and late-stage performance in each of these blocks. Performance on the no-shift sub-blocks was assessed by averaging hand deviation and RT across all four trials of each sub-block.

We typically used parametric tests (ANOVA or *t* tests) to compare across different stages or groups after checking the underlying assumptions. Wilcoxon signed-rank tests were used in place of *t* tests if the data were found to deviate from normality (assessed via Shapiro–Wilk tests). Levene’s test was used to assess homogeneity of variance required for ANOVA. If this was violated, Welch’s ANOVA was used. Sphericity violations in repeated measures ANOVAs were accounted for via Greenhouse–Geisser corrections. Cohen’s *d*, matched ranked biserial correlation and ω^2^ were used as measures of effect size for the *t* test, Wilcoxon signed-rank test and ANOVA, respectively. The significance level was set at *p* = 0.05 for all tests. Further, given the known issues with RT distributions (Wagenmakers and Brown, 2007), RT comparisons were also made using estimation statistics, which focus on the effect size and its precision. Bayesian inference methods were also used when warranted. Statistical analyses were conducted using R (version 4.0.0) and JASP (version 0.13.1).

### Data Availability

The data for all 3 experiments is freely available at: https://doi.org/10.6084/m9.figshare.19154378.v1.

## Results

In experiment 1, subjects reached to 1 of four visual targets under veridical feedback provided by means of a cursor representing hand position (Fig. 1*A*). On learning trials, the target was “jumped” counterclockwise by 45°, thereby inducing a TPE (Fig. 1*B*). Subjects were informed about the occurrence of the target-shift and instructed to reach to the new target. Interspersed within the learning block were three no-shift sub-blocks of four trials each wherein the target location was not changed and the original target stayed on the screen (no TPE). Before each of these sub-blocks, subjects were so informed and were instructed to reach to the original target. At the end of the sub-block, subjects were once again told that the target would start “jumping” and they should reach to the new target as before (Fig. 1*C*).

### TPEs stimulated intentional changes in reach direction

**Figure 2.**
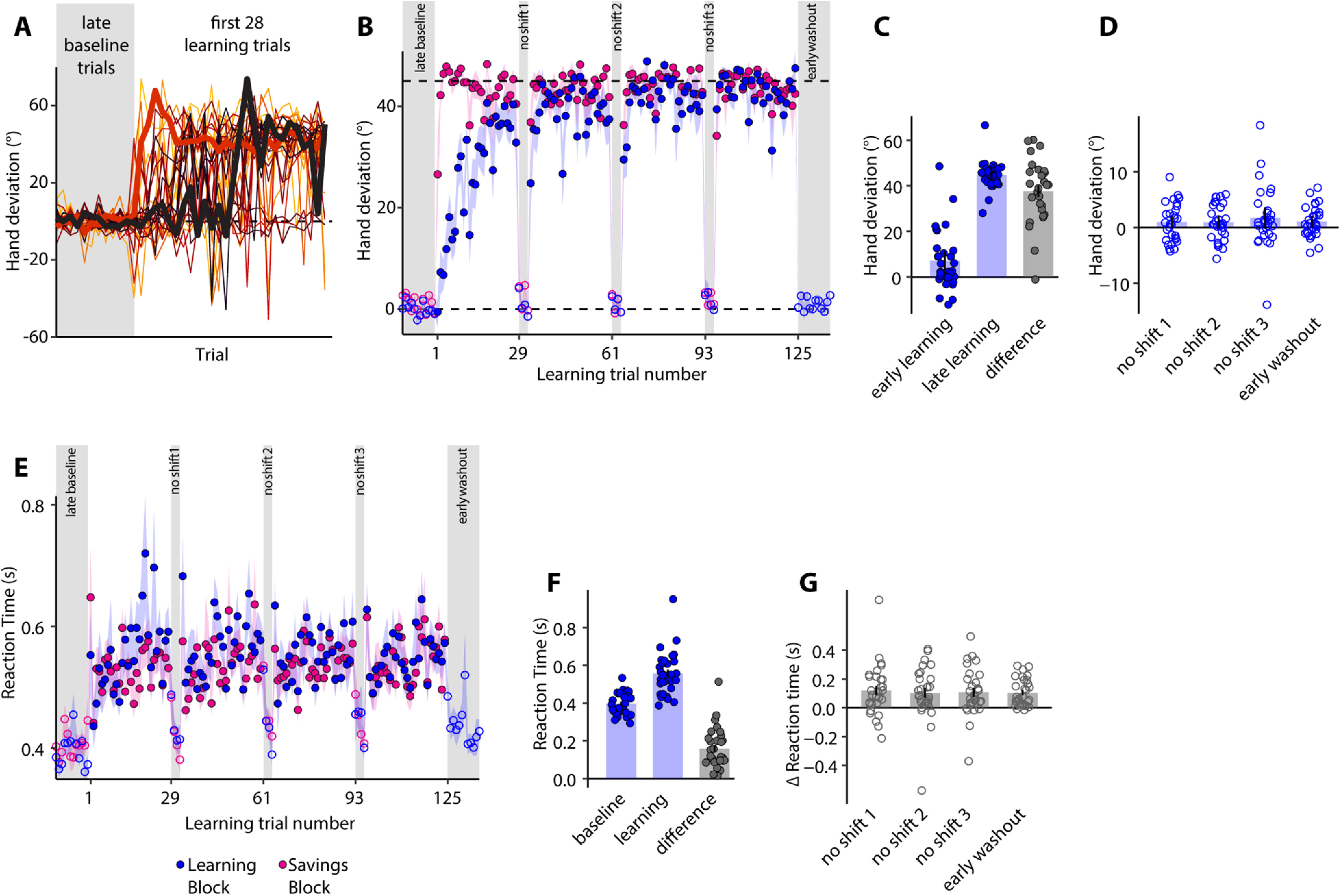
TPEs are compensated through intentional strategies. ***A***, Hand deviation (relative to the original target) on the late baseline and first 28 learning trials (each subject shown using a different color). The profile of two subjects is bolded to highlight the variability across subjects. One of them changed movement direction quite early during learning while the other did so quite late. ***B***, Group-averaged hand deviation across trials. Shaded regions denote SEM. Learning (blue) and savings (pink) data are superimposed for ease of comparison; trial 1 corresponds to the first learning trial (or the first savings trial). No-shift trials are highlighted using gray bands. Hand deviation on late baseline, no-shift and early washout trials is shown using open circles. ***C***, Mean hand deviation during early and late learning. Dots represent individual subjects. Error bars are SEM. ***D***, Mean hand deviation on the no-shift sub-blocks and early washout trials. Dots are individual subjects. Error bars are SEM. ***E***, Group-averaged RT across trials. Shaded regions denote SEM. No-shift sub-blocks are highlighted in gray. RT on no-shift trials as well as late baseline and early washout trials, is shown in open circles. ***F***, Mean RT in the baseline and learning blocks. Dots represent individual subjects. Error bars are SEM. ***G***, Change in RT on the no-shift and early washout trials relative to the immediately prior learning trial. Dots represent individual subjects. Error bars are SEM.

We first examined the change in hand angle relative to the original target direction over the learning trials. These changes were quite idiosyncratic, with some subjects showing a rapid (within a few learning trials) shift of hand direction toward the new target while others continuing to aim toward the original target for a number of trials before abruptly switching their aim toward the new target (Fig. 2*A*). Hardly any subject showed a gradual, progressive change in hand direction. The steadier trial-by-trial change in the group mean (Fig. 2*B*, blue), therefore, resulted from averaging. Differences in subject performance during the initial learning phase were also evident as highly variable hand deviations (Fig. 2*C*). Despite these early differences, all subjects learned to aim directly toward the new target location by the end of the learning block (Fig. 2*C*, mean hand deviation during the late learning stage = 44.65 ± 1.13°). Thus, subjects were able to account for the TPE and adjust their reach direction accordingly.

Performance on the no-shift sub-blocks allowed us to probe the process through which subjects learned to cancel the TPE. On these trials, subjects aimed directly toward the original target as instructed, and hand deviation fell to near zero on each of the sub-blocks (first: 0.947 ± 0.633°, 99%CI = [−0.797, 2.691], second: 0.945 ± 0.576°, 99%CI = [−0.643, 2.533], third: 1.711 ± 1°, 99%CI = [−1.044, 4.466]; Fig. 2*D*). Postlearning aftereffects were also absent with near zero hand deviation (mean ± SE = 1.022 ± 0.497°, 99%CI = [−0.349, 2.393]) on early washout trials (Fig. 2*D*). Statistically, there was no difference between the late baseline trials, no-shift sub-blocks and early washout trials (*F*_(2.656,77.022)_ = 1.219, *p* = 0.307). This immediate unlearning indicated that the change in hand angle on the target-shift trials of the learning block was because of the use of an intentional strategy that could be “turned off” on instruction.

We next predicted that if subjects were using a deliberative strategy to aim toward the displaced target on the learning trials, their RTs would be higher on those trials. We observed (Fig. 2*E*,*F*) that while baseline RT was close to 400 ms (397 ± 11 ms), it increased to ∼550 ms on the target-shift trials ( 556 ± 21 ms), a change that was clearly statistically significant (Wilcoxon signed-rank test, *W* = 0, *p* < 0.001, matched ranked biserial correlation = −1.000; estimation statistics: 95%CI of paired mean difference = [0.127, 0.203], *p* < 0.001 for two-sided permutation *t* test with 5000 bootstrap samples). Critically, on the no-shift sub-blocks, when subjects were informed that the target would not jump, their RT dropped considerably compared with the immediately prior learning trials (Fig. 2*E*,*G*). Likewise, RT on the early washout trials was smaller than the late learning trials. There was no difference in the magnitude of RT reduction across the three no-shift sub-blocks and the early washout trials (*F*_(3,87)_ = 0.1314, *p* = 0.941, ω^2^ = 0; Fig. 2*G*). This pattern, an increase in RT when the target location shifted but an immediate reduction when it did not, bolstered the view that the TPE-mediated learning on the target-shift trials was deliberate in nature.

### Savings occurred on re-exposure to TPEs

**Figure 3.**
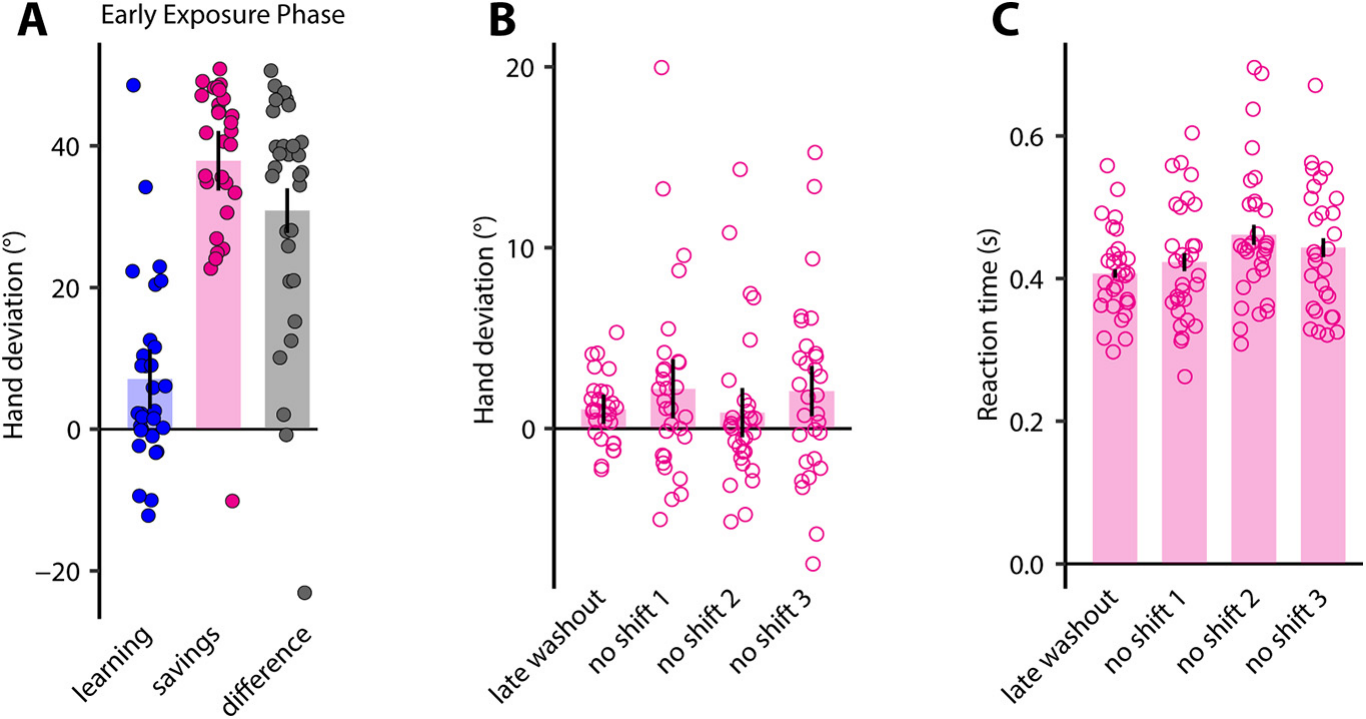
Strategy-use results in savings. ***A***, Mean hand angle during the early learning (blue) and early savings (pink) phase. Dots represent individual subjects. Error bars are SEM. ***B***, Mean hand angle on late washout and no-shift trials of the savings block. Dots represent individual subjects. Error bars are SEM. ***C***, Average RT on late washout and no-shift trials of the savings block. Dots are individual subjects. Error bars are SEM.

We next probed for savings and posited that if savings reflects the recall of learned strategies, it should occur when subjects are re-exposed to the TPEs. We found that hand angle changes from the original to the new target direction occurred over far fewer trials than initial learning, suggesting savings from prior learning (Fig. 2*B*, pink). Hand deviation was much larger during the early phase of the savings block than the learning block (Wilcoxon signed-rank test, *W* = 9, *p* < 0.001, matched ranked biserial correlation = −0.961; Fig. 3*A*). Additionally, on the no-shift sub-blocks, subjects again demonstrated rapid disengagement of learning. Hand deviation was now close to zero again (Fig. 3*B*), and there were no significant differences relative to the late washout trials (*F*_(3,87)_ = 1.167, *p* = 0.327, ω^2^ = 0.003). As was the case during learning, RT increased on the target-shift trials of the savings block, but also dropped to late washout levels on the no-shift sub-blocks (Fig. 3*C*). Collectively, the results of this first experiment indicated that in the absence of SPEs, TPEs are compensated via intentional mechanisms that are responsive to verbal instruction. The use of such strategies also promotes savings, suggesting that exposure to SPEs may not be necessary for this purpose.

### TPE-mediated changes in movement direction were verbalizable

In our second experiment (Fig. 1*D*), we sought to directly analyze how subjects explicitly formulate their reaching strategy while adapting to TPEs. Unlike experiment 1, which used an indirect, exclusion method, here we asked subjects to directly report their aiming angle on each trial with the help of a ring of equiangular numerical landmarks concentric to the start position (Taylor et al., 2014). Subjects performed reaches to targets that “jumped” 45° counterclockwise on learning trials; they were also informed about the occurrence of the jumps and instructed to reach to the new target location. On washout trials, they were again informed that the targets would not jump and they should reach to the original target.

**Figure 4.**
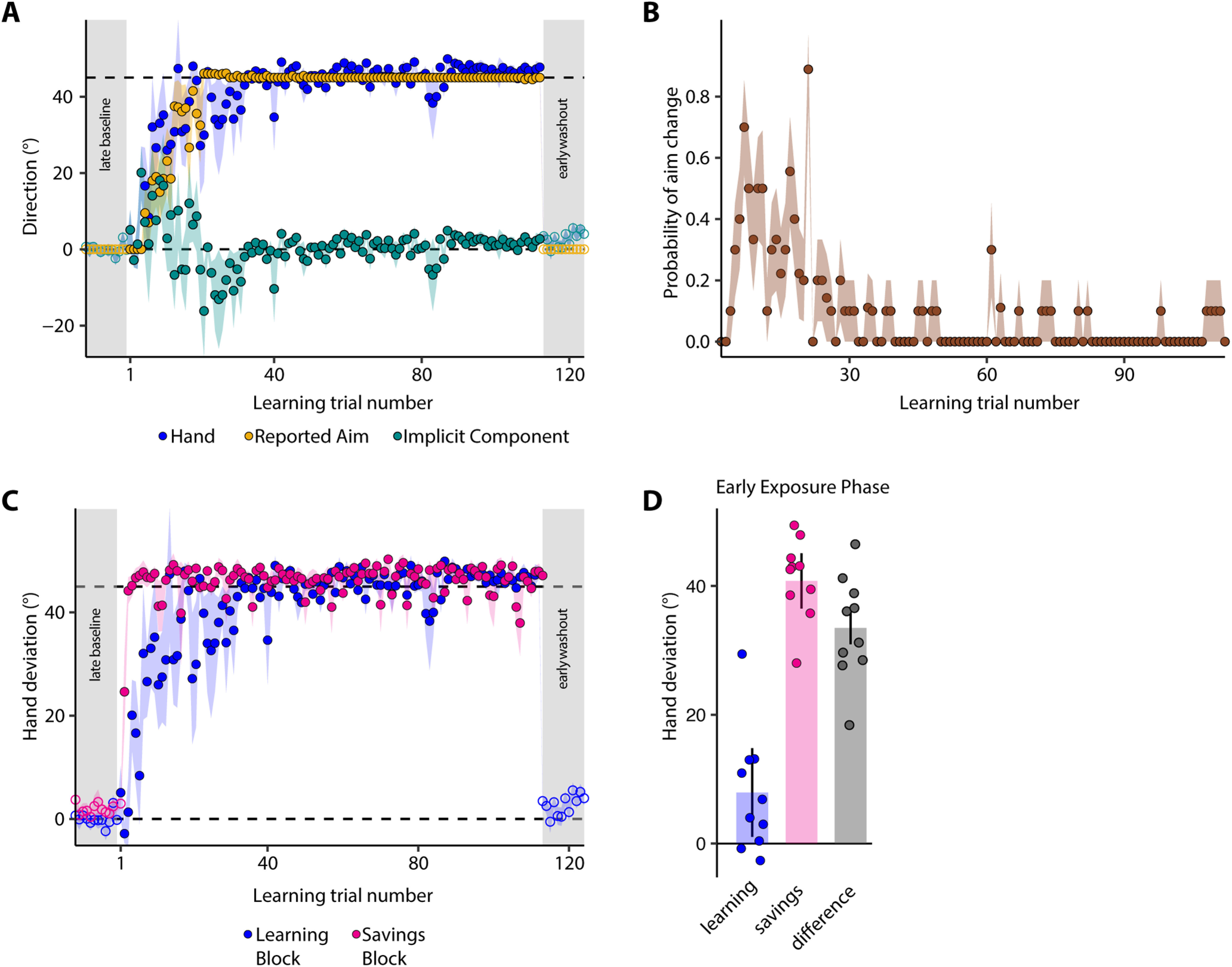
Directional changes in response to TPEs are verbalizable. ***A***, Group-averaged hand deviation (blue), reported aiming direction (yellow), and the implicit component (green) across trials. Shaded regions denote SEM. ***B***, Mean trial-wise probability of aim change across learning trials. Shaded regions are SEM. ***C***, Group-averaged hand deviation across trials. Shaded regions denote SEM. Learning (blue, same as in ***A***) and savings (pink) data are superimposed for ease of comparison; trial 1 corresponds to the first learning trial (or first savings trial). Late baseline and early washout trials are shown using open circles. ***D***, Mean hand deviation on early learning and early savings trials. Dots represent individual subjects. Error bars are SEM.

Subjects started the learning block typically by reporting landmark number “0,” which corresponded to the original target. All subjects eventually began reporting, and persisted with, their reports of the angle corresponding to the new location of the target, i.e., landmark number “9” (Fig. 4*A*, yellow). These verbal reports appeared to show higher variance during the early phase of learning, and low variance toward the end, consistent prior observations (Taylor et al., 2014). We further quantified this behavior by calculating the probability of aim change across trials of the learning block (Fig. 4*B*). This probability was much greater during the early phase of learning (reaching a peak value of ∼70% on the sixth learning trial), and dropped to ∼0 by the end of the learning block. This was also statistically confirmed as a significant difference in the aim change probability values of the early and late learning phases (Wilcoxon signed-rank test, *W* = 40.5, *p* = 0.025, matched ranked biserial correlation = 0.8)

Critically, the actual hand angle closely mirrored the reported aim. Subjects started aiming their hand (Fig. 4*A*, blue) toward the new target early on and attained complete compensation by the end of the learning block (mean = 46.194 ± 0.913°); this change was statistically robust (*t*_(9)_ = −12.116, *p* < 0.001, Cohen’s *d* = −3.831). More importantly however, there was no significant difference between the reported aiming angle and the actual hand angle at the beginning (*t*_(9)_ = 0.723, *p* = 0.488, Cohen’s *d* = 0.229) or at the end (*t*_(9)_ = 1.541, *p* = 0.158, Cohen’s *d* = 0.487) of the learning block, indicating that subjects actually aimed in the direction that they reported they would.

The difference between the reported aim and the actual hand angle provides a marker for implicit learning. We computed average implicit learning (Fig. 4*A*, green), and found that it was near zero during the early (2.744 ± 3.793°, 99% CI = [−9.581, 15.069]) as well as late (1.319 ± 0.856°, 99% CI = [−1.463, 4.101]) phases of the learning block. This indicated that subjects did not learn implicitly at all, and were using explicit strategies to compensate for the error that the target-shift induced. To confirm this, we also examined aftereffects in the washout block (Fig. 4*A*). We again found that subjects were able to immediately “unlearn” when informed that the target position would not change. Subjects not only reported landmark number “0” (corresponding to the original target location) right away, but their hand deviation on early washout trials also dropped to near zero (2.023 ± 0.858°, 99%CI = [−0.765, 4.811]). All in all, these results advocated that subjects primarily relied on the use of consciously accessible, volitional strategies to compensate for the target-shift-induced TPE.

Finally, we observed clear savings when subjects were re-exposed to the target shifts following washout. Subjects reported the new target location and also moved their hand toward it earlier (Fig. 4*C*, pink) than in the training block (Fig. 4*C*, blue). The variability in hand angle in the savings block was also low, suggesting that all subjects were able to successfully employ the previous strategy quite quickly. The change in the reported (*t*_(9)_ = −12.142, *p* < 0.001, Cohen’s *d* = −3.84) as well as actual hand angles (*t*_(9)_ = −13.223, *p* < 0.001, Cohen’s *d* = −4.182) during the early phase of the savings block were much larger compared with initial learning, indicating clearly that savings was present (Fig. 4*D*). This result once again indicated that savings does not require experience of an SPE, and is likely driven by the recall of previously employed re-aiming processes.

### Changes in reach direction were sensitive to TPE magnitude

**Figure 5.**
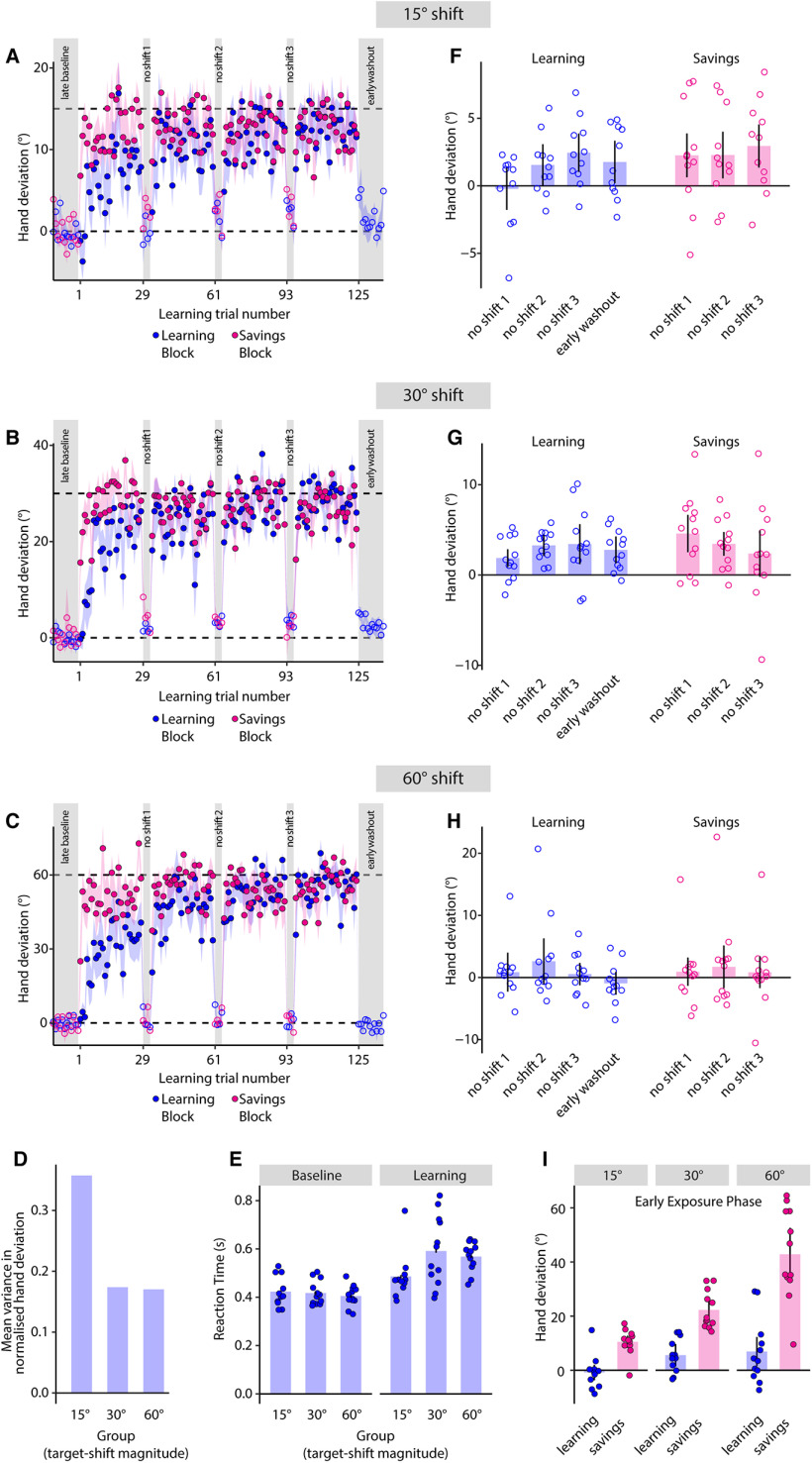
Strategies employed to compensate small versus large TPEs are likely dissociable. Group-averaged baseline-corrected hand deviation across trials for the (***A***) 15°, (***B***) 30°, and (***C***) 60° target-shift groups. Shaded regions denote SEM. Other details are same as Figure 2*A*. ***D***, Mean variance in normalized hand deviation for the three groups. No error bars are shown since this was calculated for the entire group, not individual subjects. ***E***, Mean RT on baseline and learning trials. Dots are individual subjects. Error bars are SEM. ***F*–*H***, Mean baseline-corrected hand angle on the no-shift sub-blocks of the learning block, early washout trials, and no-shift sub-blocks of the savings block for the (***F***) 15°, (***G***) 30°, and (***H***) 60° target-shift groups. ***I***, Mean hand deviation on the early learning and early savings trials. Dots represent individual subjects. Error bars are SEM.

Recent work suggests that while implicit learning is relatively rigid and insensitive to perturbation size, strategy use engenders greater flexibility (Bond and Taylor, 2015). We therefore hypothesized that the change in hand angle would scale with the size of the TPE rather than simply have a binary effect. We tested this idea in our third experiment by adopting a design similar to experiment 1 (Fig. 1*C*) but assigning subjects to three groups that differed based on TPE size (15°, 30°, or 60°). Task instructions and their schedule remained identical to experiment 1. All three groups changed their reach direction to account for the shift in target location. While hand deviation during early learning was not different between the groups (*F*_(2,32)_ = 2.609, *p* = 0.09, ω^2^ = 0.084), it was clearly so at the end of learning (15° group: 12.032 ± 2.076°, 30° group: 29.458 ± 1.426°, 60° group: 54.239 ± 2.261°, *F*_(2,32)_ = 117.274, *p* < 0.001, ω^2^ = 0.869; compare asymptote phase of Fig. 5*A–C*). This scaling indicated that the adaptive response was indeed sensitive to the size of the TPE.

### Strategies for compensating small versus large TPEs were dissociable

Interestingly, we observed that for the 15° group, the average compensation was less complete than the other groups. By the end of learning, this group had compensated only ∼80% of the TPE (mean = 80.21 ± 13.84%), while the 30° and 60° groups had compensated >90% (98.19 ± 4.75% and 90.4 ± 3.77% respectively). Importantly, this was not because subjects in the 15° group had achieved a “good enough” solution, i.e., they were able to hit the shifted target without having to fully compensate for the TPE. Considering that the target diameter was 1.5 cm, the cursor would hit the target if the hand angle changed by 12.11° for a 15° shift. However, we found that even at the end of learning, subjects did not reach this threshold on >50% of the trials (mean = 52.27%). This indicated that compensation indeed remained incomplete in this group. We additionally observed that the average variance in (normalized) hand direction during the learning block was greater following the 15° TPE (Fig. 5*D*). These patterns in the data motivated a finer analysis, wherein we probed whether the manner in which subjects responded to the small TPE (15°) differed from the larger ones (30° and 60°).

We first focused on the RT data. While RT increased on the learning trials for all groups relative to baseline, this increase was not uniform (Fig. 5*E*). We observed a dichotomous response: a small increase for the 15° group (ΔRT = 63 ± 21 ms), but larger increases for the 30° (172 ± 38 ms) and 60° (163 ± 14 ms) groups. This was statistically confirmed via a significant group difference in a Welch’s ANOVA (*F*_(2,19.144)_ = 7.702, *p* = 0.004, ω^2^ = 0.18). *Post hoc* tests revealed not only that the RT increase was much more for the 30° (*p* = 0.022) and 60° (*p* = 0.037) groups relative to the 15° group, but also that these two larger TPE groups did not differ from each other (*p* = 0.97). RT differences between the 15° and 30° groups were confirmed using estimation statistics (95%CI of unpaired mean difference = [0.024, 0.188], *p* = 0.026 for two-sided permutation *t* test with 5000 bootstrap samples), as were the differences between the 15° and 60° groups (95%CI of unpaired mean difference = [0.048, 0.144], *p* = 0.001 for two-sided permutation *t* test with 5000 bootstrap samples). Likewise, a Bayesian independent samples *t* test, which yielded a BF_10_ value of 0.38, provided support to the hypothesis that RTs of the 30° and 60° groups were not different from each other; the same was confirmed using estimation methods (95%CI of unpaired mean difference = [−0.1, 0.061], *p* = 0.663 for two-sided permutation *t* test with 5000 bootstrap samples). In sum, these patterns indicated that RT did not scale uniformly with error size.

Another hint supporting a potential dissociation in strategies for compensating small versus large TPEs came from the hand angle data of the no-shift sub-blocks, although, admittedly, this was less clear than the variability, amount of learning and RT results reported above. Consider the behavior of the 15° group first. For these subjects, we observed that the mean hand deviation on the first no-shift sub-block was close to zero (−0.222 ± 0.845°, 99%CI = [−2.901, 2.456]). However, hand deviation on the subsequent no-shift sub-blocks did not return to these levels (Fig. 5*F*). Specifically, hand deviation on the third no-shift sub-block was larger than that on the first such sub-block (*t*_(10)_ = −2.6651, *p* = 0.0237, Cohen’s *d* = −0.8036). Furthermore, the deviation on the early washout trials remained (marginally) elevated relative to the first no-shift sub-block (paired *t* test, *t*_(10)_ = −2.2265, *p* = 0.0501, Cohen’s *d* = −0.6713), but was not different from that on the last such sub-block (paired *t* test, *t*_(10)_ = 1.3732, *p* = 0.1997, Cohen’s *d* = 0.414). This suggested that there was some tendency for the learned behavior to persist even after the perturbation had been removed. Notably, this was also the case when we used baseline uncorrected data for our analyses, suggesting that this result was not an artifact of baseline bias elimination. It is however possible that some of these results were influenced by a potential outlier who showed a hand deviation of approximately −7° on the first no-shift sub-block. When this subject was excluded, the difference in hand deviation on the first and last no-shift sub-block was borderline significant with a medium-large effect size (*t*_(9)_ = −2.262, *p* = 0.05, Cohen’s *d* = −0.7154). In the Bayesian realm, the same comparison (without the outlier) yielded a BF_10_ value of 1.7627 (error = 0.0018%), which provided anecdotal evidence in favor of the hypothesis that hand deviation on the last no-shift sub-block was greater than that on the first such sub-block in this group. This difference may therefore be interpreted with some caution.

In contrast, there was clearly no difference in hand deviation between the first and last no-shift sub-blocks for the 30° (*t*_(11)_ = −1.8882, *p* = 0.0856, Cohen’s *d* = −0.5451) or 60° (Fig. 5H, *t*_(11)_ = 0.1659, *p* = 0.8713, Cohen’s *d* = 0.0479; Fig. 5*G*) groups. Likewise, we found no difference between the early washout trials and the first no-shift sub-block for the 30° group (*t*_(11)_ = −1.3371, *p* = 0.2082, Cohen’s *d* = −0.386). This was also the case for the 60° group (*t*_(11)_ = 1.2449, *p* = 0.239, Cohen’s *d* = 0.3594). This suggested that these subjects immediately and consistently returned to earlier performance levels across all no-shift sub-blocks as well as the washout block. Collectively, the distinct trends in variability, fraction of TPE compensated, and RT and hand deviation data suggested that smaller TPEs (15° in our case) might be compensated differently relative to larger ones [30°, 45° (experiment 1), and 60°].

Finally, we observed that when re-exposed to target-shifts after washout, subjects in all groups exhibited savings, as was the case in experiments 1 and 2. Subjects compensated for the imposed TPE by directing their hand toward the new target faster than they did in the training block. This expression of savings was also reliably captured via our statistical comparisons: mean hand angle was clearly larger on the early savings trials compared with the early learning trials for each group (15° group: *t*_(10)_ = −5.226, *p* < 0.001, Cohen’s *d* = −1.576; 30° group: *t*_(11)_ = −6.952, *p* < 0.001, Cohen’s *d* = −2.007; 60° group: *t*_(11)_ = −7.545, *p* < 0.001, Cohen’s *d* = −2.178; Fig. 5*I*).

Taken together, our results indicate that: (1) in the absence of an SPE, adaptive responses to consistently presented TPEs occur in the form of volitional strategies; (2) these strategies could be sensitive to the size of the TPE; and (3) strategy use facilitates savings; a history of exposure to SPEs is not needed for savings to occur.

## Discussion

In a series of experiments, we probed how the motor system responds to recurring TPEs. We demonstrate that TPEs are compensated entirely via intentional, explicitly-accessible strategies, reflecting enhanced action selection. A fundamental question is whether such compensation constitutes “adaptive” behavior at all. Insofar as adaptation is defined as a change in motor behavior following exposure to a perturbing environment, the answer is yes. However, if it is viewed more narrowly as a performance change set in motion specifically by SPEs, then perhaps no. We imposed no SPE, and the change in motor output was potentiated by a TPE elicited via a target shift.

There are many reasons to believe that this change was explicitly driven. In experiment 1, individual-level changes in hand direction were quite idiosyncratic and the group-level exponential trend emerged only as an artifact of averaging. This is not observed with implicit learning, wherein individual subjects also typically demonstrate exponential changes. Further, there was a substantial RT increase on target-shift trials, suggesting the engagement of time-consuming and deliberative mental processes (Fernandez-Ruiz et al., 2011; Haith et al., 2015; McDougle and Taylor, 2019). Subjects also disengaged from the “learned” behavior immediately on instruction, with a concomitant drop in RT; such flexibility is a hallmark of explicit but not implicit learning (Bond and Taylor, 2015). Relatedly, no aftereffects were evident on washout trials. In experiment 2, subjects were able to precisely report the aiming location and also reach there, without any implicit change in their reach direction. Finally, experiment 3 revealed that the asymptotic level of hand deviation was sensitive to TPE magnitude, unlike what has been observed with implicit learning (Wei and Körding, 2009; Kasuga et al., 2013; Morehead et al., 2017). Collectively, these observations reject the possibility that TPEs, at least as imposed through shifts in target location, are compensated implicitly. Rather, our results strongly indicate that they set in motion explicitly accessible, intentional aiming strategies.

Experiment 3 suggested the intriguing possibility that strategies employed to compensate small versus large TPEs could be distinct. Large target-shifts could be compensated in two ways. First, subjects could mentally rotate reach plans for moving to the initially presented target (Fernandez-Ruiz et al., 2011; McDougle and Taylor, 2019), underpinned by premotor and M1 circuits (Georgopoulos et al., 1989; Kosslyn et al., 1998). A key prediction of this hypothesis however is that RT should scale with perturbation magnitude, which did not bear out in our data. Additionally, mental rotation can lead to incomplete learning (McDougle and Taylor, 2019) whereas we observed more complete compensation for larger errors.

A compelling alternative then is that subjects learn to re-aim by actually learning the task structure and using it to deliberatively evaluate potential actions by mentally simulating their consequences. Specifically, actions are guided by representations of outcomes they produce given the state of the environment and what these outcomes are worth, as in model-based reinforcement learning (Dickinson and Balleine, 1994; Doya, 2000; Daw et al., 2005; Doll et al., 2012). It is known that despite being time-consuming, such goal-directed algorithms are highly flexible and can be adjusted to account for changes induced via outcome revaluation, and environment and goal changes. The longer RTs on the shift trials and the rapid, instruction-driven disengagement of the strategy on the no-shift trials, are highly in line with this notion.

In contrast to model-based control, small TPEs likely set in motion different mechanisms. When the target-shifts were small, we observed greater variability during early learning, a small undershoot during the asymptotic phase, a smaller RT increase on shift trials, and persistence of the learned behavior during the late no-shift trials (though this last result was not as clear-cut as the others). We suggest that this occurs because subjects might employ a “model-free” strategy (Kaelbling et al., 1996; Sutton and Barto, 1998) to counter small TPEs. That is, they explore the solution space for a movement that cancels the TPE and then repeat it as it leads to successful or rewarding outcomes. Such a strategy engenders higher variability initially, including a few trials on which subjects move away from the direction of the shift (Fig. 2*A*, first few learning trials). Furthermore, repetition yields robust stimulus-response associations, leading to the execution of the successful action whenever a (small) target-shift occurs. Such responses are computationally frugal, but they are also inflexible, leading to a continued expression of the learned, “habitual” behavior (Graybiel, 2008), a hint of which was seen on the late no-shift and early washout trials in the 15° shift group.

Could it rather be that adaptive responses to small TPEs (15° in our case) are driven by some kind of implicit process, like for SPEs? We posit that this is not the case. Diedrichsen et al. (2005) examined changes in motor output following exposure to a 12° error elicited either via a target-shift (TPE) or a visuomotor rotation (SPE). They reported that unlike the SPE, the TPE-mediated change did not carry signatures of implicit learning. Additionally, recent work (Oza et al., 2020) has shown that when explicitly instructed to ignore a consistently occurring 10° shift in target location, subjects are able to do so quite well. A similar sensitivity to instruction has been reported for even smaller TPEs (Tsay et al., 2021). This would not be expected from a system undergoing implicit recalibration (Mazzoni and Krakauer, 2006; Morehead et al., 2017). Finally, it has been proposed that re-exposure to a perturbing environment produces an attenuation in the implicit response, and an enhancement of the strategic component that ultimately produces savings (Avraham et al., 2021). Savings was evident in our 15° target-shift group as well; since we did not induce SPEs, this can be attributed only to a strategic process. As such, we suggest that when TPEs are small, subjects choose to aim to the new target location that gets cached or memorized with practice.

Why might strategies differ for learning from small versus large TPEs? One reason could be that model-free motor exploration can be very slow in terms of the number of attempts needed to arrive at the solution, even when the task structure is simple to learn. This strategy may therefore be functionally quite limited. When the limits of exploration are reached (i.e., when TPE magnitude is beyond tolerable levels), the sensorimotor system might abandon this strategy in favor of a new one that involves extracting as much information about the environment as possible, and selecting actions that account for changes in it. Notably, a dissociation for dealing with small versus large TPEs has been shown in studies of the behavioral (Day and Lyon, 2000; Desmurget et al., 2004; Mutha et al., 2008) and neural (Day and Brown, 2001; Desmurget et al., 2001) correlates of online, feedback-mediated motor corrections. Our results suggest that a similar dichotomy could hold for feedforward processes as well.

Our experiments also clearly brought forth savings when subjects were re-exposed to the target-shift following washout. Since we never imposed an SPE, this result indicates that a history of exposure to SPEs is likely not needed for a latent memory that facilitates faster re-learning to be expressed. This nicely converges with recent work (Leow et al., 2020) demonstrating savings even when subjects never adapt to an SPE, but are exposed to a TPE before the SPE (and the solution to cancel both is the same in hand space). Our experimental design allowed us to isolate the TPE, and its disentanglement from the SPE enabled greater certainty about the determinants of latent memories in sensorimotor learning. We suggest, in conjunction with other results (Haith et al., 2015; Huberdeau et al., 2015; Morehead et al., 2015), that SPE-specific implicit mechanisms are not a significant contributor to savings.

How do strategic processes foster savings? First, stimulus-response associations such as those formed for smaller target-shifts, could get directly cached in memory and retrieved when appropriate. Such retrieval requires less time and little cognitive effort (Logan, 1988). It is not clear whether model-based simulations of action outcomes employed to counter larger target-shifts are also cached and later retrieved without any additional planning. But, another way in which savings could emerge from model-based control is that mental simulations could be used to train a model-free process to reduce computational cost in the long run; the possibility for such an interaction has been raised before (Daw et al., 2011). This is essentially a practice-mediated transition from goal-directed to automatic, habitual behavior. Such a deliberate-to-automatic change likely explains why savings occurs even when preparation time is constrained but subjects are overtrained (Huberdeau et al., 2019).

Model-based and model-free mechanisms set in motion by large and small TPEs, respectively, could be supported by distinct neural networks. Numerous rodent studies have shown that model-free learning relies on dorsolateral striatum (posterior putamen in primates). This region is richly irrigated by inputs from sensorimotor cortex, and is essential for the formation and expression of stimulus-response associations (Yin and Knowlton, 2006; Graybiel, 2008; Devan et al., 2011). In contrast, goal-directed, model-based actions require intact processing in dorsomedial striatum (caudate and rostral putamen in primates), which receives abundant inputs from prefrontal cortical areas (Yin et al., 2005; Redgrave et al., 2010). This dissociation is evident in humans as well, with greater activation in the anterior caudate for model-based control (Tanaka et al., 2008), and caudal putamen for stimulus-response mediated behavior (Tricomi et al., 2009). Importantly, it has been shown that repeated practice leading to a shift from goal-directed to more direct stimulus-response control, is also associated with a transition in activation in rostromedial (associative) to caudolateral (sensorimotor) striatum (Jueptner et al., 1997; Lehéricy et al., 2005). In our case, such a shift toward striatal circuits supporting automaticity could occur when large TPEs are repeatedly countered. This activity could support long-term motor memories that eventually give rise to savings. Strengthening this view is the finding that Parkinson’s disease patients, who show impaired stimulus-response learning (Frank et al., 2004; Shohamy et al., 2006; Rutledge et al., 2009), also show deficient savings (Bédard and Sanes, 2011; Leow et al., 2013). When a TPE is accompanied by a limb-related SPE, a parallel network involving the cerebellum and parietal cortex is likely activated to recalibrate an internal model of the physics of the limb. How these two neural systems cooperate (or compete) to forge overall adaptive behavior should be an exciting area for future investigation.

## Synthesis

Reviewing Editor: Mark Laubach, American University Decisions are customarily a result of the Reviewing Editor and the peer reviewers coming together and discussing their recommendations until a consensus is reached. When revisions are invited, a fact-based synthesis statement explaining their decision and outlining what is needed to prepare a revision will be listed below. The following reviewer(s) agreed to reveal their identity: Jinsung Wang.

Two experts reviewed your manuscript and raised some issues that you should address before the manuscript can be considered for publication. The raw reviews are given below. Please revise your manuscript to address all points that were raised. Thank you.

One change that I would like to ask you to make is in the abstract. Please revise this sentence: “Our third experiment newly brought forth a striking dissociation of performance-error-driven strategies based on error size.” For example, “Our third experiment revealed a dissociation of performance-error-driven strategies based on error size.” I feel that your original text was a bit overstated.

Second, I would like to encourage you to consider using estimation statistics for this manuscript. As someone who also studies reaction times, and is aware of the need to use non-parametric methods to deal with the nature of RT data, I have found the approach in estimation statistics to be extraordinarily useful. The approach is a combination of exploratory data analysis and permutation statistics. Many of the figures in your manuscript suggest that you could use various methods from estimation statistics. https://www.estimationstats.com

Finally, it was not clear to me what programs you used for your statistical analyses. Perhaps I missed statements about what software you used. If not, please add details for your data analysis to the revised manuscript.

Reviewer #1

The findings reported in this manuscript provide new insights into our understanding of the nature of savings, which has been studied extensively in recent motor learning literature.

This manuscript seems to describe a study that is well conducted, and the manuscript is well written. However, I have some concerns, as stated below.

1. Regarding the task employed in this study, the target jump is obvious (esp. compared to typical visuomotor rotation of the display), thus making the task very ‘explicit’. And this manipulation, or perturbation, will not involve any SPEs related to implicit adaptive processes. In a way, this task is similar to the ‘explicit learning’ task employed by Wang et al. (2019), which was cited in the present study. I’d say that the tasks employed in the current study and also in Wang et al.’s study should be considered as typical skill acquisition, as opposed to motor adaptation, tasks. That being said, I’d argue that the term ’adaptive response’, which the authors used frequently to describe their own data (e.g., lines 449, 588), is not very appropriate. That is, there was no ’adaptation’ involved in this study. Instead, how about ’performance change’, ’the pattern of performance change’ or something like that?

2. Likewise, in line 335, the authors used the term ’deadaptation’. I don’t think the term ’deadaptation’ is appropriate either, because no ’adaptation’ really took place in this experiment. Rather, the term ’unlearning’ is more appropriate.

3. Line 482-: “We noted that hand deviation on the third no-shift sub-block was larger than that on the first such sub-block for these subjects... Furthermore, their hand deviation on the early washout trials remained (marginally) elevated relative to the first no-shift sub-block...” This was likely caused by one subject who performed quite differently than others (who showed hand deviation of approx. -7 degs, shown in fig. 5F). Even if the t tests show significant differences when the data point from this subject is removed, the overall pattern of the individual data points do not look very different across blocks. I’d say the significant differences observed here reflect a type I error.

4. Line 499-: “In sum, this pattern in the RT and hand deviation data suggested that smaller TPEs (15{degree sign} in our case) might be compensated differently relative to larger ones (30{degree sign}, 45{degree sign} (experiment 1) and 60{degree sign}).” This may not be true (see my comment above). In case the t tests show significant differences even after the data point from this subject is removed, the authors should still soften this argument substantially, given the point I stated above (i.e., mention the point I stated (e.g., possibility of a type I error), then state this argument as a possibility, rather than as a finding unambiguously supported by the data (so the readers can decide on their own)).

5. Lines 513-518: My previous comment also applies here.

6. Line 522-: “Taken together, our results indicate that: 1) in the absence of an SPE, adaptive responses to consistently presented TPEs occur in the form of volitional strategies, 2) these strategies are sensitive to the size of the TPE, and 3) strategy use facilitates savings; a history of exposure to SPEs is not needed for savings to occur.” The data support, and I agree with, the first and the third conclusions, while I disagree with the second one, based on my comment above.

7. The discussion section needs to be revised based on my comment #3.

Reviewer #2

This study aimed to test how humans learn to compensate consistent TPEs imposed in isolation from SPEs, and if they express as savings this acquired memory to compensate for TPEs when re-exposed to the learning environment. This uses a recently popular methodology (Leow et al., 2020; Tsay et al., 2021; Ranjan & Smith, under preparation; Oza et al., 2020) of using mid-movement target shifts to manipulate TPEs.

Here, the authors evoke TPEs without SPEs across all trials and measured reaction time changes during learning as a proxy measure of time-consuming deliberative processes. In three experiments, this study (1) measured implicit recalibration through no-target-hit trials interspersed during adaptation, (2) measured explicit and implicit adaptation to target-shifts by measuring aiming direction, and (3) examined whether the size of the TPEs influence the way savings is expressed.

The manuscript is very well-written, I commend the authors on the writing.

Some of the results shown here (i.e., that there is no implicit recalibration after adapting to TPEs alone, and that adaptation to TPEs can result in savings, and that adaptation to TPEs are verbalizable and therefore aptly described as explicit) are consistent with and extend previous work.

The study reports two aspects that seem more novel: (1) reaches in the no-shift blocks became more adapted in the no-shift trials for the 15 degree target shift condition but not the other larger target shift conditions, and (2) reaction times increased more with larger TPEs (30 degree and 60 degree target-shifts) than the 15 degree target shifts. The authors interpret this in terms learning of a model-free stimulus-response association for small target shifts, and a model-based learning for larger target-shifts. I have a few points to make in this regard.

First, could be be possible that the incomplete adaptation for the 15 degree group (12.032 degree or so) result from people achieving a good-enough solution (i.e., they can hit the shifted-target given the 1.5 cm target size?) I’m not sure I would necessarily overinterpret the incomplete adaptation from this result. (line 453)

Second, the authors suggested qualitatively different mechanisms for the 15 deg group than for the 30 and 60 degree group, as reaction times did not increase linearly from 15 to 30 to 60 degree target shifts. This inference came from findings that the larger TPEs from 30 degree and 60 degree target-shifts induced similar increases in reaction times: this similar increase in reaction time needs to be qualified by tests of statistical equivalence, or bayesian analyses testing the null hypothesis of no difference. It would be excellent if the authors also consider this existing literature that examined reaction times with different rotation angles (without imposing target errors) which show decidedly non-linear increases in reaction time relative to rotation angle (Neely and Heath, 2010).

Third, the framing of a lack of change in reach direction from the first to the last no-shift sub-block for the 30 degree direction is very black-and-white (p=.08, cohen’s d=0.54 in line 493 is no difference?). Indeed, I am slightly concerned about the inferences made based on a change in the 15 degree condition and a lack of change in the 30 and 60 degree conditions, based on running multiple t-tests. I understand that to infer that the between-subjects factor (shift-angle) differed from the pre- to post- adaptation stages, one should run an ANOVA with the factors shift-angle (15, 30, 60) and stages (first, last no-shift) to test this interaction (Nieuwenhieus et al., 2011, Nature Neuroscience).

Fouth: I’m worried about the baseline bias correction and its impact on estimating the no-shift behaviour particularly for the small target shift angle (15). I would be reassured if the authors directly compared the baseline reach angles to the no-shift reach angles (without bias correction): if there is indeed evidence supporting a difference, then I would be more likely to be convinced that the 15 degree condition is indeed showing some model-free component that persists in the no-shift trials . At present the evidence isn’t very convincing to me because of the way the statistical analyses were conducted.

Minor points: Figure 5 FGH should have the target-shift angle explicitly labelled on the figure. The figure legend should also be explicit in stating if these hand-deviations in all the figures are all baseline bias corrected (my best guess is that Fig 5ABC are not baseline bias corrected?)
